# *par *genes in *Mycobacterium bovis *and *Mycobacterium smegmatis *are arranged in an operon transcribed from "SigGC" promoters

**DOI:** 10.1186/1471-2180-8-51

**Published:** 2008-03-27

**Authors:** Yveth Casart, Elida Gamero, Sandra Rivera-Gutierrez, Jorge A González-y-Merchand, Leiria Salazar

**Affiliations:** 1 Laboratorio de Biología Molecular. Departamento de Biología Estructural, Instituto Venezolano de Investigaciones Científicas (IVIC), Apartado 20632, Caracas 1020-A, Venezuela; 2 Departamento de Microbiología, Escuela Nacional de Ciencias Biológicas, IPN, Mexico DF, Mexico

## Abstract

**Background:**

The ParA/Soj and ParB/Spo0J proteins, and the *cis*-acting *parS *site, participate actively in chromosome segregation and cell cycle progression. Genes homologous to *parA *and *parB*, and two putative *parS *copies, have been identified in the *Mycobacterium bovis *BCG and *Mycobacterium smegmatis *chromosomes. As in *Mycobacterium tuberculosis*, the *parA *and *parB *genes in these two non-pathogenic mycobacteria are located near the chromosomal origin of replication. The present work focused on the determination of the transcriptional organisation of the ~6 Kb *orf60K-parB *region of *M. bovis *BCG and *M. smegmatis *by primer extension, transcriptional fusions to the green fluorescence protein (GFP) and quantitative RT-PCR.

**Results:**

The *parAB *genes were arranged in an operon. However, we also found promoters upstream of each one of these genes. Seven putative promoter sequences were identified in the *orf60K-parB *region of *M. bovis *BCG, whilst four were identified in the homologous region of *M. smegmatis*, one upstream of each open reading frame (ORF).

Real-time PCR assays showed that in *M. smegmatis*, mRNA-*parA *and mRNA-*parB *levels decreased between the exponential and stationary phases. In *M. bovis *BCG, mRNA-*parA *levels also decreased between the exponential and stationary phases. However, *parB *expression was higher than *parA *expression and remained almost unchanged along the growth curve.

**Conclusion:**

The majority of the proposed promoter regions had features characteristic of *Mycobacterium *promoters previously denoted as Group D. The -10 hexamer of a strong *E. coli *σ^70^-like promoter, located upstream of *gidB *of *M. bovis *BCG, overlapped with a putative *parS *sequence, suggesting that the transcription from this promoter might be regulated by the binding of ParB to *parS*.

## Background

Partitioning systems were first characterised in low copy number plasmids of *Escherichia coli*. In general, plasmid partition modules encode two trans-acting proteins and a *cis*-acting, centromere-like DNA sequence required for partitioning [1]. *E. coli *plasmid P1 and F factor partitioning systems encode: i) homologous ATPases (ParA/SopA), characterised by a conserved 'deviant' Walker A motif [2]; and ii) site-specific DNA-binding proteins containing helix-turn-helix (HTH) motifs (ParB/SopB) [3]. The centromere-like sites, *parS *and *sopC*, are located downstream of the genes encoding the trans-acting proteins [4,5]. Chromosomal homologues of *parA *and *parB *(sometimes denoted as *soj *and *spo0J*, because of their involvement in sporulation), as well as *parS*, have been identified in a wide range of Gram-negative and Gram-positive bacteria, with the exception of certain γ-proteobacteria, including *E. coli *and *Haemophilus influenzae *[3,6]. The *par *genes are commonly arranged in an operon, whose expression is autoregulated by *par*-encoded proteins [7-9]. In numerous bacteria, chromosomal *par *genes are located upstream of the *dnaA-oriC *region [10].

Two or more 16-bp *par*S inverted repeats, with a consensus sequence 5'-TGTTNCACGTGAAACA-3, are clustered near the origin of chromosome replication (*ori*C) region [11]. In *Bacillus subtilis*, Spo0J binds to 8 of these 10 pseudo-palindromic 16-bp invert repeats *in vivo*. Furthermore, the presence of one of such site on an otherwise unstable plasmid stabilizes it in a Soj- and Spo0J dependent manner [11]. In *Streptomyces coelicolor*, 20 of the 24 *parS *sequences are packed around *oriC*, and ParB binds to many of them *in vitro *and *in vivo *[12]. Although the precise function of ParA and ParB is still unclear, it has been proposed that the recruitment of these proteins to *parS *sites may lead to the positioning of replicated chromosomal origins at opposite poles of the cell [11]. The *parAB *genes are essential for the viability of *Caulobacter crescentus *[13], whereas in *B. subtilis *[14], *Streptomyces coelicolor *[15] and *Pseudomonas putida *[16], deletion of *soj*/*parA *and *spo0J*/*parB *is not lethal. *spo0J *mutants of *B. subtilis *display defects in chromosome segregation in both vegetative and sporulating cells [14,17]. Deletion of *parAB *in *S. coelicolor *results in the production of significant numbers of anucleate spores, although no detectable defect is visible in vegetatively growing cells [15]. In *P. putida*, whose cellular division occurs only by binary fission, anucleated-cells are only observed when mutants in these genes are grown in minimal medium or as they enter into stationary phase [16,18]. The Par proteins are involved in other processes, such as chromosome replication, transcription, and a cell-cycle checkpoint that links chromosome segregation to cell division [13,19,20].

New insights about the role of Par proteins in chromosome segregation are emerging with the recent discovery of the bacterial cytoskeleton. A bacterial actin homolog, MreB, has been implicated in chromosome segregation. In the bacterial cells that have MreB, a membrane-associated coiled structure extends along the cell length [21]. In *C. crescentus*, this structure may be used for transporting *oriC *rapidly towards the cell poles. MreB may bind to DNA via ParB forming a kinetocore-like complex, which might connect the *oriC *region to the MreB coil at the membrane, and thus may actively move this region toward the cell poles [22].

Tuberculosis (TB) is a major public health problem with one-third of the world's population infected by its etiologic agent, *Mycobacterium tuberculosis*. Over two million people die from TB each year [23]. The tubercle bacilli can lie dormant for years, only to rise again when the immune system weakens due to old age, malnutrition or AIDS. *M. tuberculosis *is a non-capsulate and non-spore forming bacterium with a relatively simple life cycle. Despite the medical importance of this human pathogen, very little is known about the molecular mechanisms controlling its cell cycle.

An interesting problem in *M. tuberculosis *biology is therefore to understand how this intracellular pathogen regulates progression of its cell cycle during the stages of TB infection, including the dormant state. The dormant state may be considered in some ways analogous to sporulation, and some genes related to sporulation in *B. subtilis *and *S. coelicolor *are found in the genome of *M. tuberculosis *[24]. Nevertheless, the dormant state may also be considered a special physiological state during which mycobacteria grow slowly, but are not sporulated.

Studies based on experimentally-mapped transcriptional start sites have provided a consensus sequence for several mycobacterial promoters [25-27]. Group A includes the σ^A ^and σ^B ^*Mycobacterium *promoters, which share homology to the *E. coli *σ^70 ^consensus sequence. The Group D or "SigGC" *Mycobacterium *promoters, with -10 (C_90_R_70_C_50_C_50_M_70_S_90_) and -35 (T_90_G_50_S_80_C_50_S_90_T_30_) GC rich-hexamers, are likely to be unique to mycobacteria [27,28]. However, it is still unknown which of the 13 sigma factors described in *Mycobacterium *actually drive transcription from these promoters [26,27].

In order to understand their possible role in mycobacterial cell cycle, in this work we examined the genetic regulation of the *parA *and *parB *partitioning genes, by analysing the transcription of these genes in *Mycobacterium bovis *BCG and *Mycobacterium smegmatis*, two non-pathogenic mycobacteria, belonging respectively to the slow and fast-growing groups of the *Mycobacterium *genus.

## Results

### Nucleotide sequence of the *jag-parB *region and conservation of the *parS *sites near the chromosomal origin of replication

Analysis of the complete genome sequence indicates that the ParA and ParB proteins of *M. tuberculosis *H37Rv have high sequence identity (50–60%) with the chromosomal partitioning Soj/ParA and SpoJ/ParB proteins of *S. coelicolor*, *P. putida *and *C. crescentus *[29]. Genes homologous to *parA *and *parB *were also identified in the close relatives *Mycobacterium leprae *[29], *Mycobacterium bovis *[29] and *M. smegmatis *[30] and like in *M. tuberculosis *they are located near the chromosomal origin of replication (*oriC*).

Eight ORFs could be identified in the 6 Kb region upstream of the *dnaA *gene in *M. tuberculosis*, *M. bovis *BCG and *M. smegmatis *(see Additional file 1). All eight ORFs were divergently oriented in relation to the *dnaA *gene and included the *parA *and *parB *genes along with several other conserved genes, following a similar gene order to that found in other Gram-positive and -negative bacteria [10].

*M. tuberculosis *ParA and ParB proteins had sequences that were 99% and 100% identical to the homologous proteins in *M. bovis *BCG, and 77% and 71% identical to the homologous proteins in *M. smegmatis*, respectively. In *M. tuberculosis *and *M. bovis *BCG, the stop and start codons of *gidB, parA *and *parB *genes overlapped, suggesting that these genes could be part of a single operon. In *M. smegmatis*, the stop and start codons of *gidB *and *parA *genes overlapped, while the *parA *and *parB *genes were separated by 59 nucleotides, suggesting that promoters localized in the *parA-parB *intergenic region could initiate the transcription of the *M. smegmatis parB *gene. Lin and Grossman [8] identified a 16 bp perfect palindrome (5'-TGTTTCACGTGAAACA-3') identical to the *parS *sequence of *B. subtilis*, at two sites in the *M. tuberculosis *chromosome, located at ~1.1 Kb and ~2 Kb upstream of the *parB *gene. A Blast search of this sequence revealed that two putative *parS *sites seemed to be conserved in *M. bovis *BCG and *M. smegmatis *genomes at similar positions, 1.761 Kb and 0.9 Kb upstream of the start codon of *parB *for *M. bovis *BCG, and 1.749 Kb and 0.984 Kb upstream of the start codon of *parB *for *M. smegmatis*. No additional *parS *sequences were found in these mycobacterial chromosomes.

**Figure 1:**
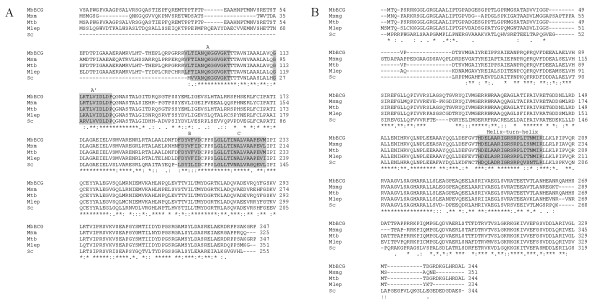
**Alignment of the ParA and ParB proteins**. Comparison of the ParA (left) and ParB (right) aminoacid sequences of *M. bovis *BCG (MbBCG), *M. smegmatis *(Msm), *M. tuberculosis *(Mtb), *M. leprae *(Mlep) and *S. coelicolor *(Sc). The alignment was carried out using CLUSTAL W (1.83) . Conserved amino acids are indicated with asterisk below the alignment; "-" represents gaps, ":" indicate conserved substitutions and "." semi-conserved substitutions. The A (VL/FTIANQKGGVGKT), A' (GLKTLVIDLDP) and B (FDYVFV/ID) boxes typical of the Walker-ATPases and the C motif (LGLLTINALVAAPEVM/L) of ParA proteins are highlighted on grey. The Helix-turn-Helix motif (HDELAARIGRSRPLITNMIR) involved in DNA-protein interactions of ParB is also highlighted on grey [3]. As noted, mycobacterial ParA have a longer N-terminal domain (between 67 to 91 aa) than other bacterial-ParA proteins.

ParA and ParB proteins alignments were performed using the translated *par *sequences proposed for *M. bovis *BCG strain Pasteur 1173P2 [29], *M. smegmatis *mc^2^155 [30], *M. tuberculosis *H37Rv [29] and *M. leprae *[29]. Multiple amino acid sequence alignments showed that all the motifs identified in the chromosomal-coding Par proteins were conserved in the mycobacterial ParA and ParB proteins (Figure 1). The high aa sequence homology at the N-terminal region of the mycobacterial ParAs – and the fact that possible RBS sequences were not identified further downstream of the proposed *parA *start codons – suggest that in contrast to other chromosome-encoded ParA proteins, mycobacterial ParAs begin far upstream of the Walker A-box motif. Therefore, the mycobacterial ParA proteins may have an unusually long N-terminal domain. However, the helix-turn-helix (HTH) DNA-binding motif present in this region of some plasmid ParA proteins homologues was not present [31].

### Promoter activity in the *parA *and *parB *regulatory regions

In order to locate the promoters responsible for the transcription of the *parA *and *parB *genes, we cloned fragments of the *orf60K-parB *region of *M. bovis *BCG and *M. smegmatis *in the promoterless vector pFPV27, upstream of the *gfp *reporter gene (Table 1). GFP stability produced the accumulation of the fluorescent protein inside the cell and therefore the fluorescence at stationary phase always was higher than at exponential phase. In addition, the absence of a transcriptional terminator upstream of the cloning site in pFPV27 resulted in a relatively high and almost constant fluorescence background during the different growth phases studied, ranging from 175 to 178 RFU. Hence, the GFP fusions performed were not to evaluate cell growth-related expression, but to identify the promoter of each gene under study. Fluorescence > 18–20 % of the background was considered to be indicative of activity of the cloned promoter(s).

**Table 1:** Plasmids used in this work

Plasmid	Relevant features	Reference or source
pFPV27	Km^r^, shuttle vector for operon and gene fusion to *gfp *gene	[46]
pD19B	261 bp PCR fragment from *M. bovis *BCG containing the upstream region of the gene *jag*	This work
pJ1B	148 bp PCR fragment from *M. bovis *BCG containing part of the coding region of the *orf60K*	This work
pA3B	114 bp PCR fragment from *M. bovis *BCG containing the upstream region of the gene *jag*	This work
pB5B	205 bp PCR fragment from *M. bovis *BCG containing the upstream region of the gene *gidB*	This work
pA15B	116 bp PCR fragment from *M. bovis *BCG containing the coding region of the gene *jag*	This work
pB3B	113 bp PCR fragment from *M. bovis *BCG containing the upstream region of the gene *gidB*	This work
pA2B	214 bp PCR fragment from *M. bovis *BCG containing the upstream region of the gene *parA*	This work
pC5B	113 bp PCR fragment from *M. bovis *BCG containing part of the coding region of the gene *parA*	This work
pE1B	229 bp PCR fragment from *M. bovis *BCG containing the upstream region of the gene *parB*	This work
pJ3M	320 bp PCR fragment from *M. smegmatis *containing the upstream region of the gene *jag*	This work
pD1M	159 bp PCR fragment from *M. smegmatis *containing the upstream region of the gene *jag*	This work
pG2M	256 bp PCR fragment from *M. smegmatis *containing part of the coding region of the gene *jag*	This work
pC18M	217 bp PCR fragment from *M. smegmatis *containing part of the coding region of the gene *parA *cloned in the direction of *parA *gene	This work
pC11M	217 bp PCR fragment from *M. smegmatis *containing part of the coding region of the gene *parA *cloned in the reverse direction of *parA *gene	This work
pA1M	120 bp PCR fragment from *M. smegmatis *containing part of the coding region of the gene *gidB*	This work
pB1M	200 bp PCR fragment from *M. smegmatis *containing part of the coding region of the gene *gidB*	This work
pB16M	475 bp PCR fragment from *M. smegmatis *containing the upstream region of the gene *parB*	This work
pC1M	122 bp PCR fragment from *M. smegmatis *containing the upstream region of the gene *parB*	This work

**Figure 2:**
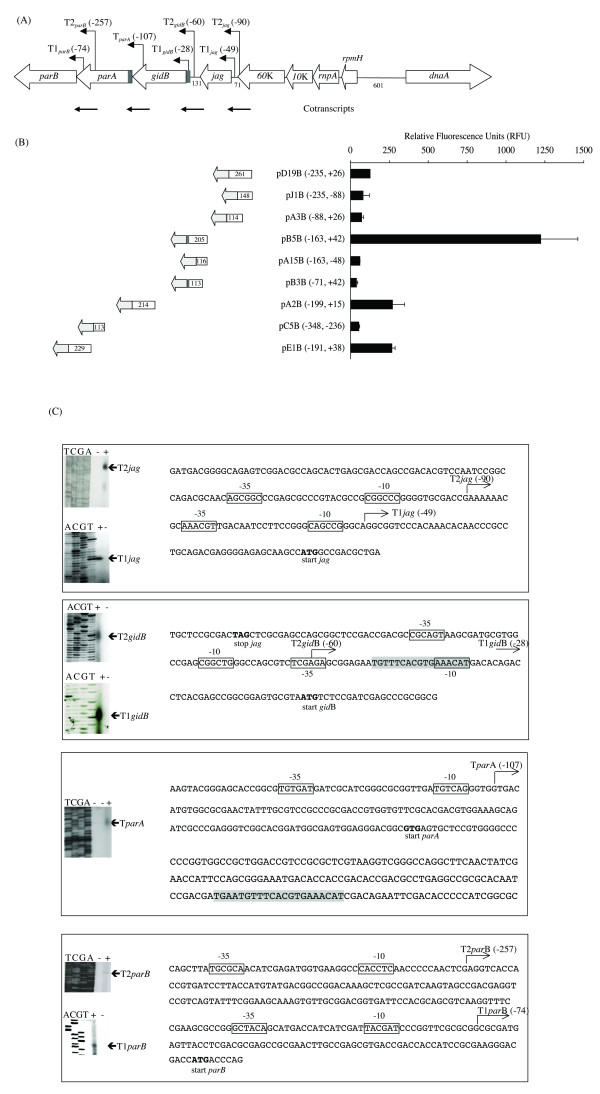
**Transcriptional pattern of the *M. bovis *BCG *orf60K-parB *region**. (A): Schematic representation of the *M. bovis *BCG *orf60K-parB *region showing the position of the transcriptional start sites (TSSs). The *parS *sequences are represented by solid grey rectangles. Cotranscripts identified by RT-qPCR are shown as horizontal bold arrows. TSSs are showed as bent arrows. The position of the TSSs mapped are in parenthesis and it localization is related to the start of the gene immediately downstream. (B): Transcriptional fusions to *gfp *and measurement of the fluorescence emission. Recombinant plasmids were obtained by cloning of PCR fragments (white rectangles) upstream of the *gfp*. The coordinates (5' and 3' ends with respect to the start codon of the gene being evaluated), of the cloned fragments are shown in parenthesis together with the plasmid name. The length (in bp) of the cloned fragments is indicated within the white rectangles and the grey arrows represent the cloning direction and the *gfp *gene. Promoter activity was measured by fluorimetry as Relative Fluorescent Units (RFU) in *M. smegmatis *corrected by subtracting pFPV27 mediated background fluorescence.The bars on the graphic represent RFU (means ± SE of at least three independently experiments) during stationary phase of growth. (C): Mapping of the mRNA 5' termini on the *jag-gidB-parA-parB *region of *M. bovis *BCG by primer extension. The mRNA 5'-ends or TSSs using specific oligos are indicated (T1*jag*, transcription start site for the promoter 1 of gene *jag*, etc.). Sequencing reaction with the same primers is shown alongside. The ParA1B primer was annealed to total RNA at 48°C. The highlighted boxed region defines the -35 and -10 promoter sequences identified upstream of each TSS; the numbers in parenthesis indicate the position to the TSS according to the start codon of the gene locate immediately downstream. Start codon for *jag*, *gidB*, *parA *and *parB *is shown in bold and the putative *parS *sequence located upstream *gidB *is highlighted with grey.

**Figure 3:**
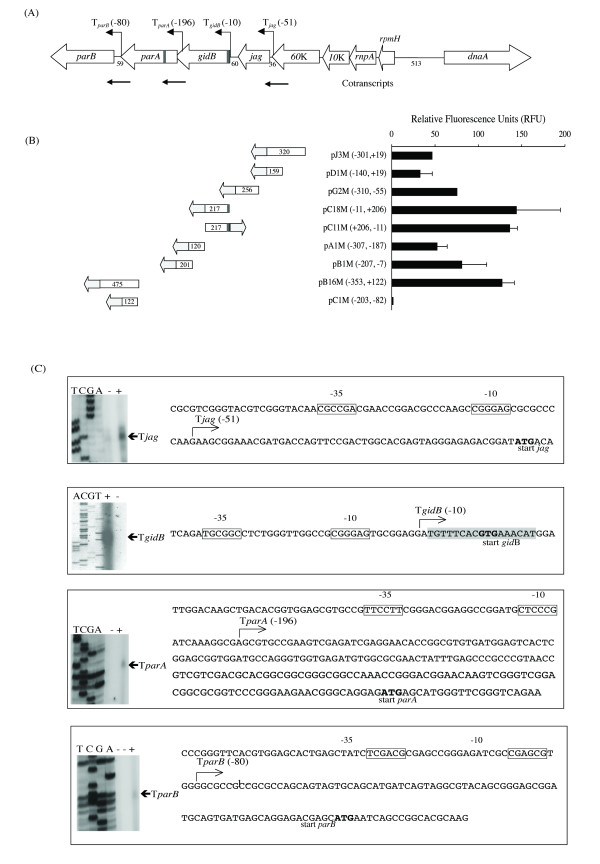
**Transcriptional pattern of the *M. smegmatis orf60K-parB *region**. (A): Schematic representation of the *M. smegmatis orf60K-parB *region showing the position of the transcriptional start sites (TSSs). The *parS *sequences are represented by solid grey rectangles. Cotranscripts identified by RT-qPCR are shown as horizontal bold arrows. TSSs are showed as bent arrows. The position of the TSSs mapped are in parenthesis and it localisation is related to the start of the gene immediately downstream. (B): Transcriptional fusions to *gfp *and measurement of the fluorescence emission. Recombinant plasmids were obtained by cloning of PCR fragments (white rectangles) upstream of the *gfp*. The coordinates (5' and 3' ends with respect to the start codon of the gene being evaluated), of the cloned fragments are shown in parenthesis together with the plasmid name. The length (in bp) of the cloned fragments is indicated within the white rectangles and the grey arrows represent the cloning direction and the *gfp *gene. Promoter activity was measured by fluorimetry as Relative Fluorescent Units (RFU) in *M. smegmatis *corrected by subtracting pFPV27 mediated background fluorescence. The bars on the graphic represent RFU (means ± SE of at least three independently experiments) during stationary phase of growth. (C): Mapping of the mRNA 5' termini on the *jag-gidB-parA-parB *region of *M. smegmatis *by primer extension. The mRNA 5'-ends or TSSs using specific oligos are indicated (T1*jag*, transcription start site for the promoter 1 of gene *jag*, etc.). Sequencing reactions with the same primers is shown alongside. The highlighted boxed region defines the -35 and -10 promoter sequences identified upstream of each TSS; the numbers in parenthesis indicate the position to the TSS according to the start codon of the gene locate immediately downstream. Start codon for *jag*, *gidB*, *parA *and *parB *is shown in bold and the putative *parS *sequence located upstream *gidB *is highlighted with grey.

All the constructs were tested for fluorescence emission in *M. smegmatis *mc^2^155. The Figures 2B and 3B show the fluorescence obtained during the stationary phase of growth for each transcriptional fusion corrected by subtracting the fluorescence emission of *M. smegmatis *bearing the plasmid pFPV27. We found that *M. bovis *BCG promoter activities were well expressed in the heterologous host *M. smegmatis*.

*M. smegmatis *cells emitted fluorescence when they bore plasmids containing the *orf60K-jag *(pD19B) and *jag-gidB *(pB5B) intergenic regions, as well as plasmids containing the 3'-end coding region of the *gidB *(pA2B) and *parA *(pE1B) genes of *M. bovis *BCG (Figure 2B), suggesting that *jag, gidB*, *parA *and *parB *genes of *M. bovis *BCG may be transcribed from promoters localised immediately upstream of each one of these genes. The *parA *and *parB *genes of *M. smegmatis *could also be transcribed from their own promoters, because substantial fluorescence was detected when the cells had the GFP transcriptional fusion to the *orf60K-jag *(pJ3M), *jag-gidB *(pG2M plasmid) and *parA-parB *(pB16Ms plasmid) intergenic regions as well as to the 3'-end of the *gidB *gene (pB1M plasmid) (Figure 3B). Unexpectedly, we found that a 217 bp fragment containing the *parS *motif localised in the 5'-end of the *gidB *gene of *M. smegmatis *(pC18Ms and pC11Ms plasmids) showed fluorescence emission independently of the clone direction, suggesting divergent promoter activity in this region.

When we deleted 89 bp of the 3'-end (pA15B) or 92 bp of the 5'-end (pB3B) from pB5B, the fluorescence emission was practically abolished, showing that the entire 205 bp region of pB5B was necessary in order to have the activity observed with this transcriptional fusion (Figure 2B). Finally, the fluorescence of *M. smegmatis *bearing some constructs (pA3B, pA15B, pB3B, pC5B, pJ3M and pG2M) was not detectable during the exponential phase of growth (data not shown), suggesting that the promoters contained in these fragments were weak and their expression could be detected only after enough GFP have accumulated during growth.

### Mapping the transcription start sites in the *jag-parB *region

In an attempt to precisely localise the transcriptional start sites (TSSs) in the *jag-parB *region of *M. bovis *BCG and *M. smegmatis*, primer extension experiments were carried out using several specific primers and total RNA isolated from exponentially growing mycobacteria (Figure 2C and 3C). Analysis of the nucleotide sequence upstream of the identified TSSs was performed in order to identify potential promoters. Published consensus promoter sequences as well as the distance between the -10 hexamer and the TSS and the length of the spacer between the -10 and -35 regions were considered. Promoter sequences proposed according to our results are shown in Table 2. All but one of the TSSs of all genes corresponded to a purine (A or G) and each one was very well associated to a recognised promoter sequence. All the identified promoters in both *M. smegmatis *and *M. bovis *BCG belonged to the Group D of *Mycobacterium *promoter recognition sequences, with the exception of two possible *E. coli *σ^70^- like promoters located upstream of *gidB *(P1*gidB*) and *parB *(P1*parB*) in *M. bovis *BCG. We found two TSSs upstream of the *jag, gid *and *parB *genes in *M. bovis *BCG (Figure 2C). They were close to each other, suggesting that two promoters may drive the expression of each one of these genes. Fragments containing only one of the proposed promoters for *jag (*pJ1B and pA3B), *gid *(pJA15 and pB3B) and *parB *(pC5B and pE1B) genes of *M. bovis *BCG showed fluorescence activity (Figure 2B) corroborating the presence of two promoters upstream of each one of these genes.

**Table 2:** Promoter sequences for *jag, gidB, parA *and *parB *genes of *M. bovis *BCG (Mb) and *M. smegmatis *(Ms)

Promoter	-35	Spacer^†^	-10	Spacer^‡^	TSS^§^	Group
P1_*jag *_(Mb)	aaaCGT	16	CAgCCG	03	A	D
P2_*jag *_(Mb)	aGCgGc	18	CGGCCC	11	G	D
P1_*gidB *_(Mb)	TcGAgA	19	aAacAT	04	C	A
P2_*gidB *_(Mb)	cGCaGT	18	CGgCtG	13	A	D
P_*parA *_(Mb)	TGtgaT	21	tGtCAG	04	G	D
P1_*parB *_(Mb)	gctACA	17	TAcgAT	12	G	A
P2_*parB *_(Mb)	TGCgCa	20	CACCtC	12	G	D
P_*jag *_(Ms)	cGCCGa	18	CGGGAG	10	G	D
P_*gidB *_(Ms)	TGCgGc	14	CGggAG	07	G	D
P_*parA *_(Ms)	TtCCtT	18	CtCCCG	10	A	D
P_*parB *_(Ms)	TcGaCg	16	CGagCG	04	G	D

^(†) ^Length of the spacer between the -35 and -10 hexamers. ^(‡) ^Length of the spacer between the -10 hexamer and TSS. ^(§) ^Transcription start site (TSS) determined by primer extension. Consensus nucleotides are shown with capital letters.

In contrast, we found just a single TSS upstream of the *jag, gid, parA *and *parB *genes in *M. smegmatis *(Figures 3A and 3C) and upstream of the *parA *gene in *M. bovis *BCG (Figure 2A and 2C). This implied the presence of only one promoter for each one of these genes.

The -10 (AAACAT) hexamer associated to the T1*gidB *of *M. bovis *BCG overlapped with a putative *parS *sequence (Figure 2C), suggesting that ParB could be regulating the transcription from P1*gidB *by competing for the same region with the RNA polymerase.

### Dicistronic transcripts in the *jag-parB *region

The primer extension, transcriptional fusions to *gfp*, and nucleotide sequence analysis together indicated that the *gid, parA *and *parB *genes of both *M. bovis *BCG and *M. smegmatis*, seem to be transcribed independently from their own promoters. However, the short or missing intergenic regions found in this study do not eliminate the possibility that *gid *and the two *par *genes can be part of a single transcript. To ascertain whether the *par *genes had a dicistronic arrangement, RT-qPCR was performed using *M. bovis *BCG and *M. smegmatis *RNAs. Specific primers were designed in order to obtain products encompassing from the 3'-end to the 5'-start of the *orf60K-jag*, *jag-gidB, gidB-parA *and *parA-parB *pair genes (Table 3), which always excluded the contribution of the promoters located immediately upstream of each evaluated gene. Although the possible presence of transcriptional termination signals into the downstream gene cannot be discarded, our results suggested that all the transcripts, except the one for *jag *gene of *M. smegmatis*, were at least dicistronic (Table 4, Figures 2A and 3A).

**Table 3:** Sequences of PCR primers used for RT-qPCR^¶^

*parAB *expression				
Gene	Forward (5'→3')	Reverse (5'→3')	Amplicon (bp)	Coordinates (5', 3')

*parA *(Mb)	aagtgttgcggacggtgattc	ggtcacgctcggcaagttc	140	+874, +1014
*parB *(Mb)	gcgtaagccgattcagatgcc	ccgagccgaactccaccac	122	+833, +954
*parA *(Ms)	acgacggccgcaccaagct	gtcgagatagctcagtgctcc	177	+754, +930
*parB *(Ms)	cgtaagccgatccagatgcca	tcgttctgggcgctcatcag	171	+882, +1052

Co-transcription				

Region	Forward (5'→3')	Reverse (5'→3')	Amplicon (bp)	Coordinates (5', 3')

*orf60K-jag *(Mb)	aatgcggcagccccaacag	tcggtggtgtcagcgtcg	256	-233, +23
*jag-gidB *(Mb)	ccagaacgccgagtcgttgtgc	gtccgaagatcgcagacgc	204	-164, +40
*gidB-parA *(Mb)	gcggttgatgtcagggtggtg	cgtcggtgtcggtggtgtc	236	-124, +112
*parA-parB *(Mb)	gcgttggagggtgtgtcg	ccctttctgcgtgacggc	352	-326, +26
*orf60K-jag *(Ms)	gctccgccaccgaactgac	gcgtccgcagcgagagtg	187	-184, +3
*jag-gidB *(Ms)	ttccgccgcctcaagcc	cacgccctgtcctttgttctg	199	-124, +75
*gidB-parA *(Ms)	atgctcccgatcaaaggc	cgaacccatgctcatctcc	230	-215, +15
*parA-parB *(Ms)	cctcgcagtgtgaaggtctcg	cggctgattcatgctcgtctcc	212	-200, +12

^¶^Normalization was performed using primers for 16S rRNA amplification previously published [50]

**Table 4:** Co-transcription in the *jag-parB *region

	Cotranscription region (cDNA copies/16S × 10^-6^)			
	*orf60K-jag*	*jag-gidB*	*gidB-parA*	*parA-parB*
*M. bovis *BCG				
Exponential (7 days)	9.16 ± 5.4	16.40 ± 1.4	17.92 ± 2.2	1.42 ± 0.1
Stationary (14 days)	43.45 ± 5.9	108.70 ± 20.4	65.85 ± 23.9	10.39 ± 0.1
				
*M. smegmatis*				
Early Exponential (OD_585nm _= 0.6)	58.27 ± 5.6	0	4.29 ± 1.2	14.95 ± 0.1
Late Exponential (OD_585nm _= 1.2)	35.61 ± 4.5	0	3.39 ± 0.5	12.36 ± 1.9
Stationary (OD_585nm _= 2.0)	0	0	0.28 ± 0.0	1.65 ± 0.1

### Quantification of *parA *and *parB *mRNA levels during mycobacterial growth

**Figure 4:**
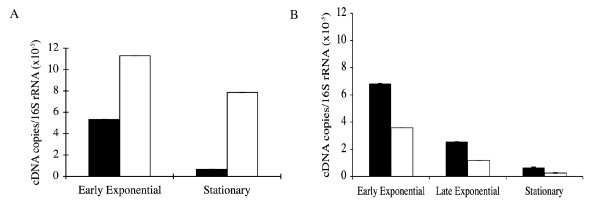
***parA *and *parB *mRNA synthesis during growth of Mycobacteria in broth culture**. (A): Total RNA isolated from exponential (7 days) and stationary (21 days) cultures of *M. bovis *BCG. (B): Total RNA isolated from exponential (OD_595 nm _= 0.9), late exponential (OD_595 nm _= 1.2) and stationary (OD_595 nm _= 3.0) cultures of *M. smegmatis*. At the indicated time, bacterial RNA was extracted and transcript levels of *parA *(black bars) and *parB *(white bars) were analysed by real-time PCR; 16S rRNA levels were used for normalization. The error bars show the mean (± SD) of at least two separate determinations made with different batches of total RNA.

The levels of *parA *and *parB *genes mRNAs in *M. bovis *BCG and *M. smegmatis *were quantified by real-time RT-PCR (RT-qPCR) in exponential as well as in the stationary growth phase. Quantitative PCRs for *parA, parB *and 16S-rRNA were performed using the cDNAs obtained from the same RT reaction. The amount of mRNA for each *par *gene was calculated and expressed in relation to the total RNA and normalized by the 16S-rRNA levels. We detected mRNA-*parA *that was double of mRNA-*parB *levels in *M. smegmatis*, although the mRNAs of both genes decreased between the exponential and stationary phases. In contrast, the mRNA-*parB *levels in *M. bovis *BCG were very similar between the exponential and stationary phases, but mRNA-*parA *levels showed an important reduction in the stationary growth phase. Additionally, unlike the transcriptional pattern observed in *M. smegmatis*, the mRNA-*parB *levels were higher that mRNA-*parA *in *M. bovis *BCG (Figure 4).

## Discussion

We found evidence that the chromosomal *parA *and *parB *genes of *M. bovis *BCG and *M. smegmatis *are expressed from multiple promoters. To identify the promoter sequences that regulate the expression of the *par *genes, we mapped the transcription start sites of the *par*-mRNAs by primer extension and confirmed the activity of the identified promoters by transcriptional fusions to a fluorescent reporter. We also demonstrated that in *M. bovis *BCG the *parA *and *parB *genes are differentially expressed during the exponential and stationary growth phases.

In all microorganisms studied thus far, plasmid and chromosome-encoded partitioning genes are arranged in an operon. Transcription of the *par *genes is driven by one (in F and R1 plasmids, P1 prophage and *C. crescentus*) or two (in *S*. *coelicolor*) promoters located upstream of the gene encoding the ATPase (*parA *or *sopA*) [5,7,13,15,32]. The *jag*, *gidB, parA *and *parB *genes of *M*. *bovis *BCG and *M. smegmatis *shared orientation and close spacing, suggesting that they may be co-transcribed. However, we identified at least one promoter sequence for each of these genes (Figures 2 and 3 and Table 2). RT-qPCR (Table 4) and Northern blot hybridisation (data not shown) demonstrated that the *parA-parB, gidB-parA and orf60-jag *gene pairs were also transcribed as dicistronic operons; however, co-transcription between the *jag-gidB *region was only detected in *M. bovis *BCG (Table 3).

Most of the putative promoter sequences identified (Table 2) had features of the *Mycobacterium *promoters denoted as Group D. Only two of the promoter sequences found belonged to Group A *Mycobacterium *promoters. We were unable to identify promoter sequences for σ factors different from σ^A ^(or σ^B^) and "SigGC" in the *jag-parB *region of both mycobacterial species, probably due to the exiguous data accumulated regarding DNA sequences recognized by RNA polymerases containing other σ factors. Nevertheless, no variation in the *parA *and *parB *gene expression has been observed in *M. tuberculosis *knockout mutants of σ^E ^[33], σ^H ^[34], σ^F ^[35], σ^C ^[36], σ^D ^[37], σ^L ^[38] or σ^M^[39], suggesting that none of these σ factors were involved in the *parAB *expression.

Based on our results, we propose that in both *M. bovis *BCG and *M. smegmatis*, the *parA *and *parB *genes comprise an operon. Therefore, the expression of *parB *may be derived from three promoters in *M. bovis *BCG – two Group D and one Group A promoters – whereas *parB *transcription in *M. smegmatis *seems to be driven from only two promoters, both belonging to the Group D of *Mycobacterium *promoters (Figures 2 and 3 and Tables 2 and 4).

Results also indicated that the *parA *and *parB *genes in *M. bovis *BCG and *M. smegmatis *were differentially expressed (Figure 4), possibly due to the differential quantity and activity that each promoter contributed to transcribe the *gidB parA *and *parB *genes in each mycobacteria. It has been suggested that mycobacterial promoters homologous to *E. coli *σ^70 ^have a higher activity than the Group D *Mycobacterium *promoters [27]. In agreement with these observations, we found that the TSSs in Group D mycobacterial promoter sequences (T2*gidB *and T2*parB*) showed weaker signals in comparison with those preceded by Group A (T1*gidB *and T1*parB*) of *Mycobacterium *promoters (Figure 2C).

The decrease of the mRNAs for *parA *and *parB *observed during the transition from exponential to stationary phase in *M. smegmatis *(Figure 3B) may be in agreement with the assumption that genes involved in replication and cell division must be down regulated during the stationary phase. In keeping with this interpretation, the expression of these genes decreases when *M. tuberculosis *is cultured under starvation [40]. The *parB *gene expression in *M. bovis *BCG seems to be differently regulated, because one Group A *Mycobacterium *promoter as well as two "SigGC" promoters appeared to contribute to *parB *expression in this mycobacterial species (Figure 2 and Table 2). The expression of *E. coli *σ^70^-like promoters (P1*parB*) appears to be particularly important for *parB*, because the transcription from P2*parB *(T2*parB *in Figure 2C) as well as from *parA *(Table 4) did not account for the mRNA-*parB *levels observed at the stationary growth phase (Figure 4A). Since during stationary growth, the levels of σ^A ^decrease [41] whilst σ^B ^expression increases [41,42], we proposed that transcription from P1*parB *may be driven by σ^B^, the principal-like sigma factor.

On the other hand, it has been suggested that the correct stoichiometry of the Par proteins is important for partition of plasmids [43,44] and the bacterial chromosome [9,45], and that therefore the *par *loci must be under strict regulation. Recently, it has been suggested that modulation of the chromosomal *parAB *expression may be mediated by the binding of ParB to *parS *sites located near promoter sequences [9]. Here, one putative *parS *site was identified in the regulatory region of the *gidB *gene of *M. bovis *BCG, which overlapped with the -10 sequence of one Group A promoter (Figure 2C), suggesting that the binding of the ParB protein to the *parS *sequence may obstruct the access of the RNA Polymerase and negatively regulate the *gidB *expression. The other putative *parS *sequence identified was located within the coding region of the *parA *gene (Figure 2A). This suggests that ParB protein may also affect the expression of the *parA *gene in *M. bovis *BCG by blocking transcription initiated from T*parA *or the translation of the mRNA-*parA*. Thus, the regulation of the *gidBparA *genes and the *parA *expression by ParB binding to the *parS *sequences might contribute to maintain appropriate levels of the Par proteins.

## Conclusion

Transcriptional analysis demonstrated that the *par *genes in *M. bovis *BCG and *M. smegmatis *had a dicistronic arrangement in which *parA *and *parB *were mainly expressed from weak "SigGC" promoters. However, additional Group A promoters were found upstream of *parB *and *gidB *in *M. bovis *BCG. Furthermore, the presence of multiple promoters for genes related to cell cycle as *parAB*, which may be regulated by different sigma factors, might be responsible of the differential regulation of these genes.

## Methods

### Media, bacterial strains and growth conditions

*E. coli *XL1-blue cultures were grown in Luria-Bertani (LB) broth or on LB agar plates at 37°C. *M. smegmatis *mc^2^155 [46] and *M. bovis *BCG Pasteur (ATCC 35734) were grown at 37°C using Middlebrook 7H9 broth or 7H10 agar supplemented with 0.5 % (v/v) glycerol and 10 % (v/v) Middlebrook OADC (Difco). To avoid clumping, Tween 80 (0.05 %) was added to liquid media. The following concentrations of antibiotics were added when appropriate: Carbenicillin (Cb, 50 μg ml^-1^) or Kanamycin (Km, 50 μg ml^-1 ^for *E. coli*, 25 μg ml^-1 ^for mycobacteria).

### Transcriptional fusion to *gfp *and fluorescence measurement

The nucleotide sequences of the *orf60k-parB *regions were obtained in a Blast search [29,30]. Fragments of variable length containing the upstream region of the genes *parA *and *parB *from *M. smegmatis *and *M. bovis *BCG were inserted into the shuttle plasmid pFPV27 [47] to obtain the transcriptional fusions to *gfp*. The fragments were the products of PCR amplification using specific primers and chromosomal DNA as template. Plasmids digestions with restriction endonucleases and sequencing confirmed the direction of the inserts. The plasmids generated (Table 1) were electroporated in *M. smegmatis *mc^2^155 and grown at 37°C in 7H9 medium containing Km. Aliquots of the cultures were taken at exponential (OD_595 nm _= 0.8 – 1.3) and stationary (OD_595 nm _> 1.6) growth phases for fluorescence measurements. Fluorescence was determined from 150 μl of culture using a fluorimeter (Tecan GENius) and the appropriate filter combinations for GFP. The specific promoter activities were expressed as relative fluorescence units (RFU) corrected by subtracting the fluorescence emission of *M. smegmatis *bearing the promoterless plasmid pFPV27.

### RNA extraction and primer extension analysis

RNA was isolated from *M. smegmatis *and *M. bovis *BCG by cell disruption as previously described [48]. For primer extension experiments, at least six synthetic oligonucleotides complementary to the mRNA strand of the upstream *jag-gidB-parA-parB *sequences were 5' end labeled with [γ-^32^P] ATP and T4 polynucleotide kinase. Each labeled primer (100 fmol) was annealed to 5–20 μg of total RNA at 52°C for 30 min. After cooling at room temperature, the primer extension reactions were carried out with AMV reverse transcriptase (Promega) at 42°C for 45 min. The extension products were separated on an 8% polyacrylamide/urea gel, alongside the sequencing reaction generated using the PCR fragments corresponding to the analysed sequence and the oligonucleotide used in the primer extension reaction as primer [49].

### Detection of mRNA by quantitative RT-PCR

Total RNA was treated with DNAseI (Promega) during 45 min at 37°C and the absence of DNA was checked before reverse transcription by PCR amplification. The number of amplicons was measured by real-time PCR using gene-specific primers and SYBR Green. A standard curve was obtained for each set of primers by performing four different PCRs in parallel, using 10-fold dilutions of known amounts of *M. bovis *BCG or *M. smegmatis *chromosomal DNA (1,000, 10,000, 100,000, and 1,000,000 theoretical copies) alongside the uncharacterized samples. The melting curve of each amplicon was determined at the end of each experiment. Each measurement was performed at least in duplicate and repeated twice using independent RNA preparations from different cultures. In each sample 500 ng (or as indicated) of RNA and 0.5 μg of random hexamers (total concentration of 1 μM) were mixed in a total volume of 12 μl, heated to 65°C for 10 min and immediately chilled in ice-water for at least 5 min. Subsequently, 1 × PCR Buffer (10 mM Tris-Cl pH 8.3; 50 mM KCl), 5 mM MgCl_2_, 40 U of RNase inhibitor (RNasin Plus, Promega), 200 U of M-MLV (Moloney murine leukemia virus; Invitrogen) or AMV (Avian myeloblastosis virus; Promega) reverse transcriptase (RT) and all four deoxynucleoside triphosphates (final concentration of 1 mM each) were added. The reverse transcription reaction was performed at 42°C for 60 min. In all cases, a duplicate sample was prepared without RT as a control to measure DNA carryover. The enzyme was inactivated by heating at 99°C for 5 minutes.

Amplifications were performed in the DNA Engine Opticon (MJ Research) with sampling during elongation. Reactions were performed in 20 μl volume consisting of 0.25 μM concentration of each primer (Table 3), 10 μl of 2 × SG1Master mix (DyNAmo SYBR Green qPCR Kit. FINNZYMES) and 2 μl of the cDNA previously obtained. A control without RT was included in each run. The samples were subjected to 40 cycles of amplification (96°C denaturation for 10 s, specific annealing temperature for 15 s and 72°C extension for 20 s) in sealed strip tubes with optical caps; followed by incubation at 72°C for 5 min. To ensure that the fluorescent levels detected were due to the amplification of a specific product, a melting curve followed the final extension step, from 60°C to 95°C, with readings every 0.2°C.

### Other molecular techniques

Digestions, ligations, filling of protruding ends and plasmid DNA isolation were performed according to standard procedures. Amplified fragments and plasmid DNAs were sequenced with USB Sequenase 2.0 (USB, Amersham) and [α-^35^S]dATP or with a dye terminator cycle sequencing kit and an ABI377 sequencer (PE Biosystem), using the appropriate primers.

## Authors' contributions

LS and YC conceived the work and participated in its design. LS and JG-M coordinated the study. YC carried out the majority of the experiments while that EG and SR-G conducted some of the transcriptional fusion and primer extension assays. LS drafted the manuscript. LS and YC edited the manuscript. All authors read and approved the final manuscript.

## Supplementary Material

Additional file 1 **Gene organization in the *parB-dnaN *region of mycobacterial chromosome**. The chromosomal gene organization is shown for *Mycobacterium tuberculosis, Mycobacterium bovis *BCG and *Mycobacterium smegmatis*. Arrows indicate gene orientations. Numbers inside of the boxes denote the size in amino acids of the predicted proteins. Numbers in bold denote the length in bp of the intergenic regions. The perpendicular black lines indicate the putative *parS *motifs. An asterisk shows the *oriC *region. Click here for file

## References

[B1] Hiraga S (1992). Chromosome and plasmid partition in *Escherichia coli*. Annu Rev Biochem.

[B2] Koonin EV (1993). A superfamily of ATPases with diverse functions containing either classical or deviant ATP-binding motif. J Mol Biol.

[B3] Yamaichi Y, Niki H (2000). Active segregation by the *Bacillus subtilis *partitioning system in *Escherichia coli*. Proc Natl Acad Sci.

[B4] Mori H, Kondo A, Ohshima A, Ogura T, Hiraga S (1986). Structure and function of the F ÿplasmid genes essential for partitioning. J Mol Biol.

[B5] Austin S, Abeles AL (1983). Partitioning of unit copy miniplasmids to daughter cells. II. The partititon regions of miniplasmid P1 encodes an essential protein and a centromere-like site at which it acts. J Mol Biol.

[B6] Gerdes K, Moller-Jensen J, Bugge-Jensen R (2000). Plasmid and chromosome partitioning: surprises from phylogeny. Mol Microbiol.

[B7] Friedman SA, Austin SJ (1988). The P1 plasmid-partition system synthesizes two essential proteins from an autoregulated operon. Plasmid.

[B8] ÿDam Mikkelsen N, Gerdes K (1997). Sok antisense RNA from plasmid R1 is functionally inactivated by RNase E and polyadenylated by poly(A) polymerase I. Mol Microbiol.

[B9] Dubarry N, Pasta F, Lane D (2006). ParABS systems of the four replicons of *Burkholderia cenocepacia *: new chromosome centromeres confer partition specificity. J Bacteriol.

[B10] Gal-Mor O, Borovok I, Av-Gay Y, Cohen G, Aharonowitz Y (1998). Gene organization in the *trxA/B-oriC *region of the *Streptomyces coelicolor *chromosome and comparison with other eubacteria. Gene.

[B11] Lin DC, Grossman AD (1998). Identification and characterization of a bacterial chromosome partitioning site. Cell.

[B12] Jakimowicz D, Chater K, Zakrzewska-Czerwinska J (2002). The ParB protein of *Streptomyces coleicolor *A3(2) recognizes a cluster of *parS *sequences within the origin-proximal region of the lineal chromosome. Mol Microbiol.

[B13] Mohl DA, Gober JW (1997). Cell cycle-dependent polar localization of chromosome partitioning proteins in *Caulobacter crecentus*. Cell.

[B14] Ireton K, Gunther NW, Grossman AD (1994). *spo0J *is required for normal chromosome segregation as well as the initiation of sporulation in *Bacillus subtilis*. J Bacteriol.

[B15] Kim HJ, Calcutt MJ, Schmidt FJ, Chater KF (2000). Partitioning of the linear chromosome during sporulation of *Streptomyces coelicolor *A3(2) involved an *oriC*-linked *parAB *locus. J Bacteriol.

[B16] Lewis RA, Bignell CR, Zeng W, Jones AC, Thomas CM (2002). Chromosome loss from *par *mutants *of Pseudomonas putida *depends on growth medium and phase of growth. Microbiology.

[B17] Sharpe ME, Errington J (1996). The *Bacillus subtilis soj-spo0J *locus is required for a centromere-like function involved in prespore chromosome partitioning. Mol Microbiol.

[B18] Godfrin-Estevenon AM, Pasta F, Lane D (2002). The *parAB *gene products of *Pseudomonas putida *exhibit partition activity in both *P. putida *and *Escherichia coli*. Mol Microbiol.

[B19] Mohl DA, Easter J, Gober JW (2001). The chromosome partitioning protein, ParB, is required for citokinesis in *Caulobacter crescentus*. Mol Microbiol.

[B20] Ogura Y, Ogasawara N, Harry EJ, Moriya S (2003). Increasing the ratio of Soj to Spo0J promotes replication initiation in *Bacillus subtilis*. J Bacteriol.

[B21] Graumann PL (2004). Cytoskeletal elements in bacteria. Curr Opin Microbiol.

[B22] Gitai Z, Dye NA, Reisenauer A, Wachi M, Shapiro L (2005). MreB actin mediated segregation of a specific region of a bacterial chromosome. Cell.

[B23] World Health Organization (WHO) Tuberculosis (TB). http://www.who.int/tb/en/.

[B24] Cole ST, Brosh R, Parkhill J, Garnier T, Churcher C, Harris D, Gordon SV, Eiglmeier K, Gas S, other authors (1998). Deciphering the biology of *Mycobacterium tuberculosis *from the complete genome sequence. Nature.

[B25] Gomez M, Smith I, Hatfull GF, Jacobs WR, Jr (2000). Determinants of mycobacterial gene expression. Molecular genetics of Mycobacteria.

[B26] Smith I, Bishai WR, Nagaraja V, Cole ST, Eisenach D, McMurray DN, Jacobs WR, Jr (2005). Control of mycobacterial transcription. Tuberculosis and the tubercle bacillus.

[B27] Unniraman S, Chatterji M, Nagaraja V (2002). DNA gyrase genes in *Mycobacterium tuberculosis *: a single operon driven by multiple promoters. J Bacteriol.

[B28] Bannatine JP, Barletta RG, Thoen CO, Andrews RE (1997). Identification of *Mycobacterium paratuberculosis *gene expression signals. Microbiology.

[B29] The new multi-microbial genome browser (GenoList) GenoList genome browser. http://genolist.pasteur.fr/.

[B30] J. Craig Venter Institute Comprehensive microbial resource: *Mycobacterium smegmatis *mc2 genome page. http://cmr.tigr.org/cgi-bin/CMR/GenomePage.cgi?org=gms.

[B31] Hayes F, Barilla D (2006). The bacterial segrosome: a dynamic nucleoprotein machine for DNA trafficking and segregation. Nature Rev.

[B32] Hayes F, Radnedge L, Davis MA, Austin SJ (1994). The homologous operons for P1 and P7 plasmid partition are autoregulated from dissimilar operator sites. Mol Microbiol.

[B33] Manganelli R, Voskuil MI, Schoolnik GK, Smith I (2001). The *Mycobacterium tuberculosis *ECF sigma factor sigmaE: role in global gene expression and survival in macrophages. Mol Microbiol.

[B34] Manganelli R, Voskuil MI, Schoolnik GK, Dubnau E, Gomez M, Smith I (2002). Role of the extracytoplasmic-function sigma factor sigma (H) in *Mycobacterium tuberculosis *global gene expression. Mol Microbiol.

[B35] Geiman DE, Kaushal D, Ko C, Tyagi S, Manabe YC, Schroeder BG, Fleischmann RD, Morrison NE, Converse PJ, Chen P, Bishai WR (2004). Attenuation of late-stage disease in mice infected by the *Mycobacterium tuberculosis *mutant lacking the SigF alternate sigma factor and identification of SigF-dependent genes by microarray analysis. Infect Immun.

[B36] Sun R, Converse PJ, Ko C, Tyagi S, Morrison NE, Bishai WR (2004). *Mycobacterium tuberculosis *ECF sigma factor *sigC *is required for lethality in mice and for the conditional expression of a defined gene set. Mol Microbiol.

[B37] Raman S, Hazra R, Dascher CC, Husson RN (2004). Transcription regulation by the *Mycobacterium tuberculosis *alternative sigma factor SigD and its role in virulence. J Bacteriol.

[B38] Dainese E, Rodrigue S, Delogu G, Provvedi R, Laflamme L, Brzezinski R, Fadda G, Smith I, Gaudreau L, Palù G, Manganelli R (2006). Posttranslational regulation of *Mycobacterium tuberculosis *extracytoplasmic-function sigma factor σ ^L ^and roles in virulence and in global regulation of gene expression. Infect Immun.

[B39] Agarwal N, Woolwine SC, Tyagi S, Bishai WR (2007). Characterization of the *Mycobacterium tuberculosis *sigma factor SigM by assessment of virulence and identification of SigM-dependent genes. Infect Immun.

[B40] Betts JC, Lukey PT, Robb LC, McAdam RA, Duncan K (2002). Evaluation of a nutrient starvation model of *Mycobacterium tuberculosis *persistence by gene and protein expression profiling. Mol Microbiol.

[B41] Manganelli R, Dubnau E, Tyagi S, Kramer FR, Smith I (1999). Differential expression of 10 sigma factor genes in *Mycobacterium tuberculosis*. Mol Microbiol.

[B42] Voskuil MI, Visconti KC, Schoolnik GK (2004). *Mycobacterium tuberculosis *gene expression during adaptation to stationary phase and low-oxygen dormancy. Tuberculosis.

[B43] Funnel BE (1988). Mini-P1 plasmid partitioning: excess ParB protein destabilizes plasmids containing the centromere *parS*. J Bacteriol.

[B44] Kusukawa N, Mori H, Kondo A, Hiraga S (1987). Partitioning of the F plasmid: overproduction of an essential protein for partition inhibits plasmid maintenance. Mol Gen Genet.

[B45] Figge RM, Easter J, Gober JW (2003). Productive interaction between the chromosome partitioning proteins, ParA and ParB, is required for the progression of the cell cycle in *Caulobacter crecentus*. Mol Microbiol.

[B46] Snapper SB, Melton RE, Mustapha S, Kieser T, Jacobs WR (1990). Isolation and characterization of efficient plasmid transformation mutants of *Mycobacterium smegmatis*. Mol Microbiol.

[B47] Valdivia RH, Hromockyj AE, Monarck D, Ramakrishnan L, Falkow S (1996). Applications for the green fluorescent protein (GFP) in the study of host-pathogen interactions. Gene.

[B48] Salazar L, Guerrero E, Casart Y, Turcios L, Bartoli F (2003). Transcription analysis of the *dnaA *gene and *oriC *region of the chromosome of *Mycobacterium smegmatis *and *Mycobacterium bovis *BCG, and its regulation by the DnaA protein. Microbiology.

[B49] Movahedzadeh F, Gonzalez-Y-Merchand JA, Cox RA, Parish T, Stoker NG (2001). Transcription start-site mapping. Mycobacterium tuberculosis protocols, Methods in Molecular Medicine.

[B50] Shi L, Jung YJ, Tyagi S, Gennaro ML, North RJ (2003). Expression of Th1-mediated immunity in mouse lungs induces a *Mycobacterium tuberculosis *transcription pattern characteristic of nonreplicating persistence. Proc Natl Acad Sci.

