# Supplementary figures and images for: par genes in Mycobacterium bovis and Mycobacterium smegmatis are arranged in an operon transcribed from "SigGC" promoters
Source: BMC Microbiol. 2008 Mar 27;8:51. doi: 10.1186/1471-2180-8-51 (PMC2346475; doi:10.1186/1471-2180-8-51)

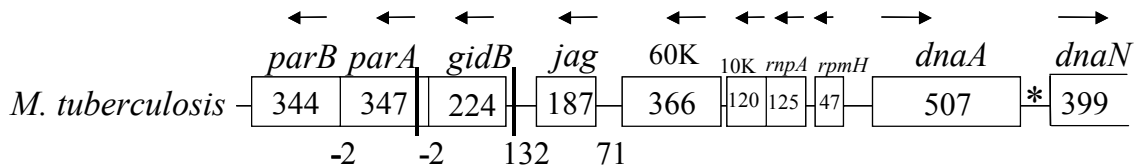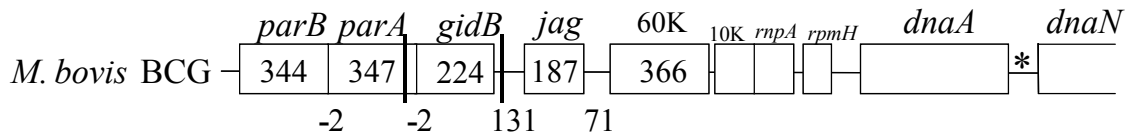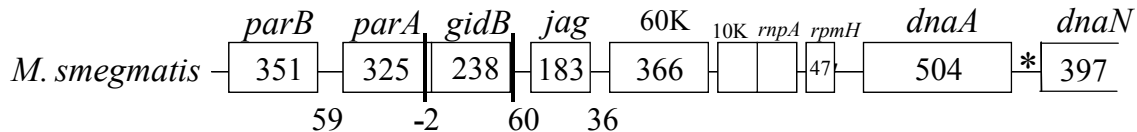

Supplement: Additional file 1 — Gene organization in the parB-dnaN region of mycobacterial chromosome. The chromosomal gene organization is shown for Mycobacterium tuberculosis, Mycobacterium bovis BCG and Mycobacterium smegmatis. Arrows indicate gene orientations. Numbers inside of the boxes denote the size in amino acids of the predicted proteins. Numbers in bold denote the length in bp of the intergenic regions. The perpendicular black lines indicate the putative parS motifs. An asterisk shows the oriC region. [file 1471-2180-8-51-S1.pdf]
